# Imagining What Could Have Happened: Types and Vividness of Counterfactual Thoughts and the Relationship With Post-traumatic Stress Reactions

**DOI:** 10.3389/fpsyg.2018.00515

**Published:** 2018-04-20

**Authors:** Ines Blix, Alf Børre Kanten, Marianne Skogbrott Birkeland, Siri Thoresen

**Affiliations:** ^1^Norwegian Centre for Violence and Traumatic Stress Studies, Oslo, Norway; ^2^Department of Psychology, Faculty of Social Sciences, University of Oslo, Oslo, Norway; ^3^Bjørknes College, Oslo, Norway

**Keywords:** post-traumatic stress, counterfactual thinking, trauma, PTSD, cognition

## Abstract

A growing body of research suggests that counterfactual thinking after traumatic events is associated with post-traumatic stress reactions. In this study we explored frequency of upward and downward counterfactuals in trauma-exposed individuals, and how trauma-related counterfactuals were represented in terms of vividness. We examined the relationships between vividness and frequency of counterfactual thoughts and post-traumatic stress reactions in two groups who had experienced different types of traumatic exposure, namely survivors and bereaved from the fire on the ferry Scandinavian Star in 1990. Even after 26 years, both survivors and bereaved reported that they currently entertained thoughts about what could have happened during the fire on Scandinavian Star. Survivors reported more downward counterfactuals than the bereaved, whereas the bereaved reported more upward counterfactuals than the survivors did. Vividness of counterfactual thoughts, as well as reported frequency of upward and downward counterfactuals, were associated with post-traumatic stress reactions. Our results suggest that both upward and downward counterfactuals can be harmful, and that vivid counterfactuals about a traumatic event might play a similar role in post-traumatic stress as trauma memories. Therefore, traumatized individuals who entertain counterfactual thoughts may benefit from interventions that target these thoughts specifically.

## Introduction

Common reactions to traumatic events are intrusive memories or thoughts, numbing, hyper-arousal and avoidance of trauma reminders, trauma-related thoughts and memories. These responses can be understood as normal responses to an abnormal event, and they subside for most individuals during the following days or weeks. However, sometimes these reactions persist, and some individuals go on to develop enduring symptoms of post-traumatic stress disorder (PTSD) (American Psychological Association [APA], 2013). According to theories on post-traumatic stress, patterns of cognitive processing of the traumatic event influence the development and maintenance of post-traumatic stress symptoms (Brewin et al., 1996; Ehlers and Clark, 2000). Along these lines, trauma has been associated with rumination and intrusive vivid memories (Ehlers and Clark, 2000; Berntsen et al., 2003; Michael et al., 2007). Interestingly, recent research has shown that some victims of trauma are not only preoccupied with what actually happened, but also with thoughts about “what could have happened” (Teigen and Jensen, 2011). In fact, a growing body of research suggests that thoughts about counterfactual outcomes play a key role in the experience of psychological distress and symptoms of PTSD (El Leithy et al., 2006; Blix et al., 2016; Mitchell et al., 2016).

Counterfactual thinking (CFT) refers to the mental simulation of alternatives to reality. An event can be mutated “upwards” to simulate a better alternative (e.g., if I had worn a helmet, I would have been fine), or “downwards” to a less desirable alternative to reality (e.g., If I had not used a helmet, I would have been more hurt). According to Roese and Epstude (2017) counterfactual thoughts are typically activated by negative experiences, or more generally by factors related to goal blockage (e.g., perception of a problem, disconfirmed expectancy). In addition, outcomes that are avoided or missed by small margins are powerful triggers of CFT, as opposed to outcomes that were nowhere close to occurring (Meyers-Levy and Maheswaran, 1992; Markman and Tetlock, 2000). Illustrative of this phenomenon, Roese and Olson (1996) found that a missed flight scenario elicited a higher number of counterfactuals when the protagonist was only five rather than 60 min late (see also Kahneman and Miller, 1986). In terms of relative frequency, upward counterfactuals tend to outnumber downward counterfactuals to a considerable extent, presumably because they in most (but not all) situations provide greater functional value (e.g., by providing information on the causes of misfortune; Roese and Olson, 1995, 1997; Nasco and Marsh, 1999; Roese and Epstude, 2017).

Decades of research has shown that comparisons often lead to contrast effects (e.g., Helson and Kozaki, 1968; Herr, 1986; Mussweiler, 2003). For instance, a small and light backpack may feel particularly light after wearing a much larger and heavier one. The same principle often applies to comparisons between factual and counterfactual outcomes (Roese, 1997). Thus, the consideration of how events such as accidents, breakups, and deaths could have been avoided, can serve as an amplifier of negative affect (Miller and McFarland, 1986; Markman et al., 1993; Roese, 1994). Conversely, downward counterfactuals can provide comfort and self-enhancement by pointing to how an unfavorable outcome could have turned out even worse (White and Lehman, 2005). Feelings of regret, guilt, and relief are examples of everyday experiences that are intrinsically associated with the act of comparing reality with counterfactual outcomes (Roese, 1994; Mandel and Dhami, 2005; Van Dijk and Zeelenberg, 2005).

If CFT typically influence affective experiences via contrast effects, it is upward rather than downward counterfactuals that should be associated with distress. Several studies support this claim (e.g., Branscombe et al., 2003; Callander et al., 2007; Gilbar and Hevroni, 2007; but see Dalgleish, 2004). For instance, Gilbar et al. (2010) studied a sample of physically injured survivors of various terrorist attacks and found that upward but not downward counterfactuals were associated with a diagnosis of PTSD. However, in a recent study of how CFT related to post-traumatic stress reactions among individuals directly and indirectly exposed to the 2011 bombing of the governmental quarters in Oslo, Blix et al. (2016) found associations between both forms of CFT and post-traumatic stress reactions, and that downward counterfactuals were generally more frequent than upward counterfactuals (see also Teigen and Jensen, 2011). To our knowledge, this is the only study that has documented a link between downward counterfactuals and higher levels of post-traumatic stress reactions.

Blix et al.’s (2016) finding that downward counterfactuals were associated with more post-traumatic stress reactions resonates badly with idea that CFT tends to lead to affective contrast effects (thinking of how things could have been worse should lead one to feel better). However, Markman, McMullen and their colleagues have demonstrated that assimilation as well as contrast effects can follow from CFT (McMullen, 1997; Markman and McMullen, 2003; Markman et al., 2007). According to Markman and McMullen (2003), assimilation ensues whenever contextual features promote an experiential (as opposed to an evaluative) mode of thinking in which the perceiver simulates the counterfactual as if it was a real event. As an example of one these features, Markman and Tetlock (2000) suggest that the attention grabbing nature of perceptually salient close-call counterfactuals produces assimilation effects by default. As an illustration, it makes sense that a man who barely escapes a high-rise fire with multiple casualties, not only will experience relief over being alive but also a significant amount of negative affect as result of vivid simulations of ending up badly injured or dead. Thus, in situations where trauma victims were close to a disastrous fate, downward counterfactuals may have the ability to affect post-traumatic stress reactions much in same manner as trauma memories (cf. mnemonic model of PTSD by Rubin et al., 2008). One aim of the present study was to corroborate the claim that both downward and upward counterfactuals can be associated with symptoms of PTSD, by replicating Blix et al.’s (2016) findings with a different sample of traumatized individuals.

The simulation of how events in one’s life could have turned out better or worse may be supported by the same basic mechanisms as episodic recollection (Van Hoeck et al., 2013; De Brigard, 2014; Özbek et al., 2017). In line with this, De Brigard et al. (2013) demonstrated that the recall of personal episodes and the construction of their counterfactual counterparts involve activation of common brain regions. Thus, to understand the relationship between CFT and post-traumatic stress, it might be useful to draw on what we already know about the role of memory in post-traumatic stress. First, intrusive memories of the traumatic event have been identified as an important factor in the development and maintenance of post-traumatic stress reactions (Rubin et al., 2008). Second, Berntsen et al. (2003) reported evidence suggesting that individuals high in post-traumatic stress have more vivid memories of the traumatic event in terms of sensory impressions and degree of emotional reliving than people who score low on post-traumatic stress.

Thoughts about what might have happened may be equally disturbing as trauma memories, and have a similar impact on post-traumatic stress reactions. Indeed, in line with research on memory and distress (Rubin et al., 2008), Blix et al. (2016) have recently shown that the rate of intrusive counterfactuals is also associated with post-traumatic stress reactions. However, no study has investigated the phenomenological characteristics of trauma-related counterfactuals, more specifically how these thoughts are experienced. Thus, if CFT influences post-traumatic stress reactions in a similar fashion as memories, then we might, based on Berntsen et al.’s (2003) findings, expect that there would be an association between counterfactual vividness and post-traumatic stress reactions. To investigate this hypothesis was the second aim of present study.

Taken together, our goal was to describe the frequency of type (upwards versus downwards), and vividness of trauma-related CFT, and to examine the relationships with post-traumatic stress reactions after exposure to a real-life traumatic event. We explored these relationships in two groups with different kinds of traumatic exposure, namely survivors and bereaved from the fire on the ferry Scandinavian Star in 1990. At the time of the fire, 482 individuals were on board the ship, of whom 159 (33%) died. The fire developed very quickly, and survival depended largely on the individual’s location on the ship at the time of the fire. The majority of the bereaved (86%) lost a close family member (58% of the sample lost one and 27% lost more than one close family members). The data in the present study were collected in 2016, 26 years later.

We expected the pattern of upward and downward counterfactuals to be different across survivors and bereaved participants. Specifically, as many of the survivors were first-hand witnesses to people suffering worse fates, and had arguably found themselves in a “close-call” situation, these individuals were expected to report more downward counterfactuals than the bereaved group. Furthermore, we anticipated the bereaved group, for whom it should be harder to imagine how things possibly could have been worse, to report more upward counterfactuals than the survivor group.

In terms of the relationship between CFT and PTSD, we predicted that both upward and downward counterfactuals would be associated with post-traumatic stress reactions among the survivors. For the bereaved group, our primary prediction was that upward counterfactuals would predict post-traumatic stress reactions (as we expected this group to report low levels of downward counterfactuals).

In line with knowledge about trauma and memory (Berntsen et al., 2003), we hypothesized that counterfactual vividness would be associated with higher levels of post-traumatic stress reactions. We operationalized vividness with several questions directed to the degree to which counterfactual simulation felt like experiencing a real event (e.g., whether CFT involved a sense of smell). As more vivid simulations should be associated with stronger emotional reactions (Markman and McMullen, 2003), we also included a question in the vividness measure about the extent to which CFT engendered intense emotions.

## Materials and Methods

### Participants and Procedure

The present cross-sectional study was a part of a systematic investigation of the current mental health status of Norwegian survivors and bereaved requested by the Norwegian Parliament in 2016 (Thoresen et al., 2017). Based on lists over survivors and bereaved that we received from an independent commission appointed by the Norwegian Parliament with the mandate to evaluate the Scandinavian Star case, we contacted the 163 Norwegian survivors and 158 bereaved during the autumn of 2016, 26 years after the fire. Information about the study and an invitation to participate were sent by post, and those who did not want to participate were given the possibility to notify us if they did not wish to be contacted on the telephone. The interviewers made contact by phone and asked if they wished to participate. Informed consent was a two-step process. Before the interview, the participants (*N* = 193) consented in writing that the information could be included in our report to the Norwegian Government. After completing the interview, most participants (96%, *N* = 185) gave an additional written consent to use the information for research purposes. In total, 185 individuals participated in the present study, including 94 survivors and 91 bereaved, resulting in a response rates of 57 and 58%, respectively. The survivors included both passengers and employees at the ship. The bereaved included individuals who were not present on the ship, but who lost someone close. Data collection took the form of face-to-face interviews, combined with questionnaires filled out by the participants on a tablet with the interviewer available. The responses were transferred encrypted to storage within the University of Oslo’s services for sensitive data (TSD). To ensure confidentiality, no data were stored on the tablet. The data used in the current study were part of the self-report section of the interview. Strict procedures were followed to ensure confidentiality, and the study was approved by the Regional committee for medical and health research ethics.

### Measures

#### Frequency of Upward and Downward CFT

For the purpose of the present study we developed a questionnaire to measure the frequency of upward and downward CFT during the last month. The participants were given the following instruction “Think back on the thoughts you have had about Scandinavian Star. Using the scale below, please indicate how often during the last month you have had thoughts as described below,” and were asked to respond to three statements about downward CFT and three statements about upwards CFT using five-point scales ranging from 1 = never to 5 = very often. **Downward:** *“I think about how much worse things could have been; I think about how much worse it could have turned out; I imagine scenarios where the outcome is worse”* and **Upward:** *“I think about how much better things could have been; I think about how much better it could have turned out; I imagine scenarios where the outcome is better.”*

#### Vividness of Trauma-Related CFT

Participants were instructed to write down their most prominent and frequent counterfactual thought related to the fire on Scandinavian Star: *“After dramatic events it is not uncommon to imagine how things might have been different. People sometimes say “if…..” What is your most frequent “If …” thought regarding the fire on Scandinavian Star*?” Subsequently, participants were asked to take a moment to reflect on this thought, and answer six questions pertaining to experienced vividness and intensity on a scale from 1 (not at all) to 7 (to a very large extent) (the individual test items are presented in **Table 2**). Unfortunately, a substantial number of participants [44 participants (24%) omitted to write down the counterfactual they had in mind, but answered the questions about how they experienced these thoughts in terms of vividness. Nine participants (5%) answered that they did not have “what if” thoughts regarding the Scandinavian Star].

#### Post-traumatic Check List (PCL)

The PCL-5 is a 20 items self-administered questionnaire that assesses DSM-5 PTSD symptoms (Blevins et al., 2015). We used the PCL-S (specific), and the items were specifically linked to the fire on Scandinavian Star. The participants were asked to indicate on five-point scales the extent to which extent they had been bothered by each symptom during the last month. The average PCL symptoms score is used in the regression analyses in the present study. A cut off score of 31 has been shown to predict PTSD (Blevins et al., 2015). Cronbach’s alpha for the total scale was 0.94.

### Confirmatory Factor Analyses

In order to test the measurement models of the new scales of Upward and Downward CFT as well as vividness of CFT, we performed confirmatory factor analyses (CFAs). Values of RMSEA less than 0.05 and values of CFI above 0.95 were considered to signify a well-fitting model (Browne and Cudeck, 1992; Hu and Bentler, 1999). An analysis with Upward and Downward counterfactuals modeled as two correlated latent factors showed an acceptable model fit: χ^2^(8, *N* = 184) = 15.051, *p* = 0.058, CFI = 0.985, RMSEA = 0.069. This indicates that these constructs are different from each other, even though they correlate moderately (*r* = 0.21, *p* < 0.005). Furthermore, a CFA with the six items on vividness and intensity loading on one factor showed good model fit: χ^2^(9, *N* = 180) = 13.563, *p* = 0.138, CFI = 0.985, RMSEA = 0.053, indicating that these items measured one common construct.

## Results

Distribution of gender, mean age, mean years of education after secondary school, and mean PCL scores are presented separately for survivors and bereaved (**Table 1**). While the groups did not differ in age or years of education, or gender, the bereaved group reported higher levels of post-traumatic stress symptoms. Among the bereaved 19% (17 out of 91) of the participants scored above cut-off for a PTSD diagnosis according to their responses on the PCL. Among the survivors 16% (15 out of 94) scored above cut-off.

**Table 1:** Mean participant characteristics with standard deviation in parentheses.

Characteristics	Survivors (*n* = 94)	Bereaved (*n* = 91)	
		
	*n* (%)/mean (*SD*)	*n* (%)/mean (*SD*)	
Gender			
Female	43 (46)	51 (56)	χ^2^(1) = 1.96, *p* = 0.18
Male	51 (54)	40 (44)	
Age in years	53.4 (14.4)	57.4 (14.5)	1.85, *p* = 0.07
Years of education (post-secondary school)	3.3 (1.4)	3.4 (1.4)	0.76, *p* = 0.46
PCL	0.70 (0.71)	0.95 (0.83)	2.17, *p* = 0.03

*Group comparisons are reported as chi-square and *t*-tests with associated *p*-values. PCL, post-traumatic check list*.

To investigate differences in vividness of CFT between survivors and bereaved, independent sample *t*-tests were performed (**Table 2**). Mean vividness scores did not differ between groups.

**Table 2:** Mean scores on the counterfactual vividness items with standard deviations in parentheses.

*“When I think about what could have happened …”*	Survivors	Bereaved	*t, p*
It is like I can hear what is happening	2.31 (1.98)	2.52 (2.03)	0.70, *p* = 0.49
It is like I can see what is happening	3.16 (2.28)	3.39 (2.15)	0.68, *p* = 0.50
It is like I can smell what is happening	2.90 (2.32)	2.17 (1.93)	-2.22, *p* = 0.03
It is like a real episode	2.92 (2.20)	2.98 (2.17)	0.15, *p* = 0.86
I can feel intense emotions	3.15 (2.21)	3.50 (2.14)	1.07, *p* = 0.29
It is like traveling in time	2.66 (2.14)	2.68 (2.12)	0.07, *p* = 0.94
Total counterfactual vividness scores	2.87 (1.90)	2.88 (1.71)	0.04, *p* = 0.97

### Frequency of Upward and Downward CFT in Survivors Versus Bereaved

The majority of the survivors (75%) as well as the bereaved (85%) reported at least some current upward or downward counterfactual thought related to the ferry disaster 26 years ago.

A mixed model ANOVA with Type of CFT (upward vs. downward) as the within factor and Group (survivors vs. bereaved) as the between factor, indicated a significant main effect of Type, *F*(24,1) = 35.30, *p* < 0.001, $\eta_{p}^{2}$ = 0.17, but this effect was qualified by a significant interaction between Type and Group *F*(42,1) = 61.20, *p* < 0.001, $\eta_{p}^{2}$ = 0.26. Follow-up *t*-tests showed that the bereaved reported a higher frequency of upward CFT (Mean = 2.68, *SD* = 1.21) compared to the survivors [Mean = 1.88, *SD* = 1.02, *t*(182) = 4.83, *p* < 0.001]. The survivors reported a higher frequency of downward CFT (Mean = 2.05, *SD* = 1.04) compared to the bereaved [Mean = 1.46, *SD* = 0.75, *t*(87) = 9.83, *p* < 0.001]. The bereaved participants reported a higher frequency of upward compared to downward CFT. For the survivors there was no significant difference in frequency of downward compared to upward CFTs [*t*(92) = -1.32, *p* = 0.19]. There was no main effect of group, *F*(179,1) = 0.53.01, *p* = 0.47, $\eta_{p}^{2}$ = 0.003.

### CFT and Post-traumatic Stress Reactions

To test whether associations between PCL scores and aspects of CFT (upward CFT, downward CFT, and vividness) depended on group (survivors vs. bereaved), we initially conducted three separate multiple regression analyses each specifying a single aspect of CFT, Group, and their interaction as predictors, and PCL scores as the criterion (variables were centered prior to analyses). None of the interaction effects turned out significant. Thus, we proceeded with a multiple regression analysis with the four predictors (upward, downward, vividness, and group) entered in the same equation, controlling for age and gender. This analysis showed that higher frequency of downward CFT (*b* = 0.17, *SE* = 0.06, β = 0.20, *p* < 0.005), higher frequency of upward CFT (*b* = 0.22, *SE* = 0.05, β = 0.34, *p* < 0.001), and higher level of counterfactual vividness (*b* = 0.10, *SE* = 0.03, β = 0.23, *p* < 0.005), were independently associated with higher PCL scores. There was no significant effect of group (*b* = -0.12, *SE* = 0.11, β = 1.08, *p* = 0.27).

## Discussion

Even after 26 years, both survivors and bereaved reported that they currently entertained thoughts about what could have happened during the fire on Scandinavian Star. The present results contribute to existing research by demonstrating that vividness of counterfactual thoughts, in addition to reported frequency of counterfactuals, is associated with post-traumatic stress reactions. Furthermore, we add to the scant literature demonstrating that downward as well as upward counterfactuals can be associated with post-traumatic stress reactions (Blix et al., 2016).

Given the fact that the fire occurred almost three decades earlier, it might seem surprising that survivors and bereaved still report considerable levels of current trauma-related CFT. It is possible, however, that the vivid nature of trauma-related counterfactuals can help to explain this finding. Specifically, research in social cognition has demonstrated a link between vividness and cognitive availability in the sense that more vivid information tends to be more easily remembered (Reyes et al., 1980; Shedler and Manis, 1986). Thus, in the context of CFT, vivid “what if” thoughts might entail a level of availability that makes them more resistant to the passage of time than counterfactuals that are more mundane. Counterfactuals that involve sensory impressions across smell, hearing, and vision might also bring the traumatic event subjectively closer in time, reinforcing the availability of trauma-related thoughts. In addition, the association between vividness and availability might help to explain why trauma-related counterfactuals often are experienced as intrusive and involuntary (Blix et al., 2016).

The present study is the first to demonstrate directly that the relative frequency of distress inducing upward vs. downward counterfactuals depends on the type of traumatic exposure. In line with earlier research suggesting that negative experiences tend to trigger upward counterfactuals (Roese and Epstude, 2017), the bereaved reported mainly thoughts about how things could have turned out better. For the survivors however, there is an extra variable at play; they were not only victims of a tragic event, but also “lucky” (Teigen and Jensen, 2011) in the sense that they survived a fire that easily could have claimed their lives. Thus, unlike the bereaved, these individuals had faced a level of closeness to worse outcomes that probably made downward counterfactuals particularly salient (Roese and Olson, 1996; Markman and Tetlock, 2000). Many of these individuals were also firsthand witnesses to others that did not survive, a factor that should make downward counterfactuals even more impinging (e.g., “it could have been me”). In addition, the high prevalence of downward counterfactuals in this group may be facilitated by the so-called magnitude effect in judgments of subjective closeness, which states that the subjective thresholds for perceiving counterfactual outcomes as “close” tend to be more liberal for more severe and dramatic events (Kanten and Teigen, 2015).

Although there were differences in reported frequencies of upward and downward CFT between the groups, there were no differences in the associations between CFT and post-traumatic stress. Thus, in the context of post-traumatic stress, rate of counterfactual thoughts may be more important than the type of CFT. However, different mechanisms might underlie these associations. Specifically, as both forms of CFT were positively associated with post-traumatic stress, the present findings are consistent with contrast effects for upward counterfactuals and assimilation effects for downward counterfactuals. Thus, while the former suggests a comparative thinking style in which counterfactuals are used as a standard to evaluate reality, the latter is more in line with an experiential “as if” type of thinking (see Markman and McMullen, 2003), implying a simulation style more akin to the way people process trauma memories.

Theories of post-traumatic stress hold that rumination and intrusive trauma memories are important for explaining the development and maintenance of post-traumatic stress reactions (Brewin et al., 1996; Ehlers and Clark, 2000; Rubin et al., 2008). Along these lines, CFT after trauma may be conceptualized as a form of rumination (Michael et al., 2007) that is maladaptive in terms of recovery from PTSD. Previous research has shown that rumination is associated with reduced cognitive control (Beckwé et al., 2014), and is followed by negative affect, and also impairs problem solving, interferes with instrumental behavior, and erodes social support (Nolen-Hoeksema et al., 2008).

Furthermore, the present results also show that trauma-related counterfactuals can be represented as vivid episodic thoughts that involve a form of mental time travel to what might have been. Mirroring research on trauma and memory (Berntsen et al., 2003) higher degrees of vividness of trauma-related counterfactuals were associated with higher levels of post-traumatic stress reactions. Thus, we argue that vivid counterfactuals about a traumatic event might play a similar role in post-traumatic stress as trauma memories. Importantly though, our data does not inform on whether the role of vivid counterfactuals in relation to post-traumatic stress is more important for downward assimilation effects or upward contrast effects. In addition, the present design does not permit any clear conclusions regarding whether vivid counterfactuals precede post-traumatic stress reactions or contribute to maintaining them. Although we primarily have hypothesized vivid counterfactuals as a driver of post-traumatic stress, we do suspect that vivid counterfactuals (irrespective of direction) can be a cause as well as a consequence of distress after trauma.

This is the first study that has investigated how counterfactual vividness relates to post-traumatic stress reactions. Future longitudinal and prospective studies are needed to explore the mechanisms underlying the association between upward and downward CFT, vividness of these thoughts and post-traumatic stress reactions. Furthermore, the present results rely on thoughts constructed in an interview setting and we cannot be sure to what extent these reflect CFT in daily life. Future studies should address this by investigating spontaneous CFT, for example in diary studies. There is a number of factors that we have not addressed in the present study that potentially may have affected the level post-traumatic stress reaction and/or the relationship between CFT and post-traumatic stress, for example other traumatic events, personality, mental disorders, or social support. Metacognitive capacity (Lysaker et al., 2015) are closely related to post-traumatic stress symptoms, and future studies should address the role of metacognitive capacity in the relationship between CFT and post-traumatic stress. Time is an important factor that is both strength and a limitation in this study. At the time of data collection, 26 years had passed since the fire on Scandinavian Star, and this was the only time of measurement. Hence, we cannot determine how the relationship between CFT and post-traumatic stress might have evolved over time. Still, the long period of time that had passed since the traumatic event clearly suggests that the relationship between CFT and post-traumatic stress is a persistent one.

The present findings have important clinical implications. The close link between thoughts about what could have happened and post-traumatic stress even decades after the traumatic event indicates that these thoughts should be specifically targeted in interventions. It is important to note, that both upward and downward CFT seems equally harmful. As suggested by De Brigard and Hanna (2015), practicing on emotion regulation during episodic CFT might be a successful therapeutic strategy. Furthermore, in trauma-focused therapies, it might be beneficial to work with CFT in a similar way as with the trauma narrative. Psycho-educative techniques and normalization might also lessen negative affect produced by trauma-related CFT. Future research should address whether interventions aimed at reducing trauma-related CFT can reduce symptoms of post-traumatic stress.

## Ethics Statement

This study was carried out in accordance with the recommendations of Regional committee for medical and health research ethics, with written informed consent from all subjects in accordance with the Declaration of Helsinki. The protocol was approved by the Regional committee for medical and health research ethics, REC South East.

## Author Contributions

IB developed the research questions, the CFT questionnaires, participated in the data collection, performed the analyses, and participated in the write-up of the manuscript. AK developed the research questions, and participated in the write-up of the manuscript. MB participated in developing the research questions, participated in the data collection, and in the write-up of the manuscript. ST was project leader of the systematic investigation of the current mental health status of Norwegian survivors and bereaved from the fire on the Scandinavian Star, requested by the Norwegian Parliament in 2016, and participated in developing the research questions, participated in the data collection, and in the write-up of the manuscript.

## Conflict of Interest Statement

The authors declare that the research was conducted in the absence of any commercial or financial relationships that could be construed as a potential conflict of interest.
